# Trends in Gender, Ethnicity and Less-Than-Full-Time Training among Reconstructive Plastic Surgery Registrars and Consultants between 2009 and 2020

**DOI:** 10.1016/j.jpra.2024.06.003

**Published:** 2024-07-02

**Authors:** Fawz Kazzazi, Danny Kazzazi, Dilip Gosall, Diana Kazzazi, Thomas Hedley Newman, James Stephen Arthur Green, Nicola Bystrzonowski, Gurjinderpal Pahal

**Affiliations:** a Group for Academic Plastic Surgery, Department of Plastic and Reconstructive Surgery, The Royal London Hospital, Whitechapel Road, London, United Kingdom; b University College London Medical School, 74 Huntley St, London, WC1E 6DE; c Stonyhurst College, Clitheroe, Lancashire, BB7 9PZ; d Royal Victoria Infirmary, Queen Victoria Rd, Newcastle upon Tyne, United Kingdom; e Department of Urology, Kings College Hospital, London, London, UK; f Department of Urology, Whipps Cross University Hospital, Barts Health NHS Trust, London, UK

**Keywords:** Training, Diversity, Gender, Ethnicity, Less Than Full Time

## Abstract

**Objective:**

This study aimed to examine the trends in gender, ethnicity and less-than-full-time (LTFT) training in reconstructive plastic surgery from 2009 to 2020 in the UK by comparing them to overall surgical specialties.

**Methods:**

We analysed NHS Digital workforce data from 2009 to 2020 by examining trends in gender, ethnicity and LTFT working among reconstructive plastic surgery consultants and registrars and comparing them to overall surgical specialties. Data were analysed using linear regression models.

**Results:**

The percentage of female reconstructive plastic surgery consultants and registrars increased significantly over the period, with reconstructive plastic surgery groups having higher percentages of females than overall surgical specialties. LTFT working trends varied, with declining trends among consultants and increasing trends among registrars. Ethnicity trends were complex, varying between different ethnic categories and between consultants and registrars, but showing an increase in diversity within the workforce.

**Conclusion:**

The demographics of reconstructive plastic surgeons in the UK became more diverse from 2009 to 2020 with trends indicating that this will continue in the future. However, there were significant variations among the different groups and levels of seniority, suggesting the need for targeted interventions to promote diversity and inclusivity in surgical specialties.

## Introduction

The National Health Service (NHS) is currently viewed as ‘more diverse than at any other point in history’.^1^ Understanding the growing diversification is crucial to fostering inclusivity, equity and retaining talent. Diversity includes not just gender and ethnicity, but the emergence of less-than-full-time (LTFT) training pathways as a significant adaptation towards the modernisation of surgical training. This study presents an examination of the trends in gender, ethnicity and LTFT training within the field of reconstructive plastic surgery in the UK from 2009 to 2020.

It is important to establish these trends to understand the progress and compare the speciality to others. According to a UK-based cross-sectional survey published in 2022, doctors tended to overestimate the progress made towards gender equality in medicine.^2^ The same study found that the recruitment of female doctors to several specialties has failed to keep pace with their representation in medicine in general in the UK. However, this is not the case in reconstructive plastic surgery. In Baroness Helena Kennedy's landmark diversity report for Royal College of Surgeons, she noted that reconstructive plastic surgery has one of the highest proportions of female doctors-in-training at 39.1% in 2020.^3^ This is consistent with a recent cross-sectional survey conducted for trainees that found near gender parity within reconstructive plastic surgery.^4^ Despite this reassuring data for registrars, there remain great concerns about the poor representation of females in senior and academic posts within the specialty.^5^ Therefore, it is important to understand the state of gender parity within reconstructive plastic surgery in the UK and trends that will determine the future of the specialty.

Unlike gender parity, there is a paucity of information focusing on ethnicity and LTFT within this specialty. Despite comprehensive cross-sectional studies on workforce distribution and attributes by the British Association of Plastic and Reconstructive Surgeons in 2021, the available data fall short with regard to the multi-year trends for ethnicity and LTFT—particularly for trainees. This is reflected in poor trainee understanding of the information about LTFT.^4^ For purposes of this study, LTFT covers ‘any arrangement with reduced working hours for doctors’ and common reasons for this include family, health or non-medical activities.^6^

This study intends to fill the gaps currently present in the literature. We aimed to encourage dialogue that can guide policy development, improve training programmes and make reconstructive plastic surgery even more inclusive.

## Methods

### Data Collection

Data were obtained from NHS Digital in 2022 through a ‘freedom of information’ request. It was provided as Hospital and Community Health Service workforce statistics for surgical specialties for 2009 to 2020. This information was collected by NHS Digital from multiple sources including Electronic Staff Records, censuses, Health Education England and the General Medical Council. Data were provided for all grades and specialties in the doctor workforce, but analysis was only performed on ‘Specialty Registrar’ and ‘Consultant’ as these were complete datasets. Within these grades, analysis was conducted on ‘Plastic Surgery’ and ‘All Surgical Specialties’—the former group referred exclusively to NHS Reconstructive Plastic Surgeons in the UK (not including private, aesthetic or cosmetic surgeons) and the latter included data for all nine of the other surgical specialties within the NHS.

### Terms and Categories

NHS Digital collects gender data using the labels ‘male’ and ‘female’. Typically, these labels are employed to denote an individual's sex assigned at birth. However, in the context of demographic data collection, these terms are often used to indicate a person's gender. Given that the data we examined, and most of the related literature, employ demographic data that uses ‘male’ and ‘female’ as gender identifiers rather than ‘man’ and ‘woman’, we adhered to this convention. Therefore, when we refer to ‘female’ surgical consultants or registrars, we are referring to ‘individuals who identified as female when asked about their gender’, not ‘individuals assigned the female sex at birth’.

The ethnicity data included self-declaration as: ‘Asian or Asian British’, ‘Black or Black British’, ‘Chinese or Chinese British’, ‘Mixed’, ‘White’, ‘Other’ and ‘Unknown’.

### Data Analysis

The dataset was formatted with all values converted to percentages. Statistical analysis was conducted using the GraphPad (Prism 10) software. Tables for each categorical variable were generated to compare ‘Plastic Surgery’ with ‘All Surgical Specialties’ by year. Linear regression analyses were conducted using the ‘simple linear regression’ tool on the software. This generated multiple results for each table including: best-fit values, goodness of fit tests, individual slope significance, comparisons of slopes and comparison of elevations. The Spearman rank tests showed the variables are not normally distributed and linear regressions were suitable. Logistic regression models provided similar results but did not provide insight to trend. The p-values generated were nominal as no adjustment was been made for multiple comparisons.

Under each variable, ‘Reconstructive Plastic Surgery Consultants’ and ‘Specialty Registrars’ were compared with their ‘All Surgical Specialties’ counterparts over time.

## Results

### Data Description

Complete data were available for the period from 2009 to 2020 for ‘Consultants’ and ‘Specialty Registrars’ in ‘Plastic Surgery’ and ‘All Surgical Specialties’. Values provided were for the number of people and were converted into percentages for comparison. A summary of the analysis results can be found in Table 1. Groups were tabulated and statistical analysis was conducted. The gradient of linear trends was calculated (increasing or decreasing) and assessed to check if it was a good fit (goodness-of-fit-test: 0 = poor, 1 = perfect); an f-test was then performed to check the significance of the linear relationship. The linear trend was compared between ‘Plastic Surgery’ and ‘All Surgical Specialty’ groups for each grade. Firstly, the slopes of the linear trends were compared to understand if the rate of increase/decrease were similar between groups. Secondly, the elevation of the trend was assessed to check if the percentages were different (i.e. higher or lower). If the rate of the slope was significantly different between the groups, it was not possible to assess significance because two lines could probably cross at some point in time.

**Table 1:** Summary of Results.

Characteristic	Plastic Surgery Trend (Gradient)	Confidence Interval (C.I.)	Can plastic surgery be modelled with linear regression? (Spearman Rank; F-test)	Goodness-of-Fit for plastic surgery (R squared, 0 = poor, 1 = perfect)	All surgical specialties trend (Gradient)	C.I.	Can all surgical specialties be modelled with linear regression? (Spearman Rank; F-test)	Goodness-of-Fit for all surgical specialties (R squared, 0 = poor, 1 = perfect)	Plastic surgery slope compared to “all surgical specialities” (Are they increasing/decreasing at the same rate?)	If no to previous column, describe relationship:	Plastic surgery proprtions compared to “all surgical specialities” (Are the results of plastic surgery higher or lower?)
**CONSULTANTS**											
**Female**	Increasing (0.67)	0.54 to 0.80	Yes (p<0.0001)	0.93	Increasing (0.61)	0.58 to 0.63	Yes (p<0.0001)	0.997	Yes (p=0.27)		Higher (p<0.0001)
**LTFT**	Decreasing (-0.047)	-0.29 to 0.20	No (p=0.68)	0.017	Decreasing (-0.12)	-0.30 to 0.05	No (p=0.15)	0.19	Yes (p=0.58)		Higher (p<0.0001)
**LTFT after 2015**	Increasing (0.50)	-0.39 to 1.38	No (p=0.19)	0.38	Increasing (0.45)	0.27 to 0.62	Yes (p=0.0019)	0.93	Yes (p=0.8784)		Higher (p<0.0001)
**Asian or British Asian**	Increasing (0.12)	-0.047 to 0.29	No (p=0.14)	0.21	Increasing (0.75)	0.67 to 0.83	Yes (p<0.0001)	0.98	No (p<0.0001)	All surgicalspecialties increasing at higher rate.	Lower (cannot assess)
**Black or British Black**	Decreasing (-0.042)	-0.16 to 0.076	No (p=0.064)	0.06	Decreasing (-0.018)	-0.033 to -0.0034	Yes (p=0.021)	0.43	Yes (p=0.65)		No difference (p=0.56)
**Chinese or British Chinese**	Decreasing (-0.017)	-0.11 to 0.078	No (p=0.70)	0.16	Increasing (0.02)	0.010 to 0.030	Yes (p=0.001)	0.67	Yes (p=0.40)		Higher (p=0.002)
**Mixed**	Increasing (0.24)	0.16 to 0.33	Yes (p<0.0001)	0.8	Increasing (0.10)	0.084 to 0.12	Yes (p<0.0001)	0.94	No (p=0.0015)	Plastic surgery increasing at higher rate.	Higher (cannot assess)
**White**	Decreasing (-0.37)	-0.62 to -0.13	Yes (p=0.007)	0.53	Decreasing (-0.91)	-0.97 to -0.85	Yes (p<0.0001)	0.99	No (p=0.0001)	All surgical specialties decreasing at higher rate.	Higher (cannot assess)
**Other**	Increasing (0.035)	-0.11 to 0.18	No (p=0.61)	0.027	Inceasing (0.031)	-0.0094 to 0.071	No (p=0.12)	0.23	Yes (0.95)		No difference (p=0.37)
**REGISTRARS**											
**Female**	Increasing (1.7)	1.34 to 2.11	Yes (p<0.0001)	0.91	Increasing (1.01)	0.93 to 1.09	Yes (p<0.0001)	0.99	No (p=0.0007)	Plastic surgery increasing at a higher rate	Higher (cannot assess)
**LTFT**	Increasing (0.36)	0.17 to 0.55	Yes (p=0.0017)	0.64	Increasing (0.25)	0.18 to 0.31	Yes (p<0.0001)	0.89	Yes (p=0.22)		No difference (p=0.74)
**LTFT after 2015**	Increasing (0.67)	-0.045 to 1.38	No (p=0.06)	0.63	Increasing (0.37)	0.27 to 0.48	Yes (p=0.0007)	0.96	Yes (p=0.29)		No difference (p=0.43)
**Asian or British Asian**	Decreasing (-0.029)	-0.74 to -0.34	No (p=0.83)	0.048	Decreasing (-0.54)	-0.33 to 0.27	Yes (p=0.0001)	0.79	No (p=0.0046)	All surgical specialities decreasing at a higher rate.	Lower (cannot assess)
**Black or British Black**	Increasing (0.048)	0.033 to 0.25	Yes (p=0.0154)	0.46	Increasing (0.027)	0.14 to 0.26	Yes (p<0.0001)	0.84	Yes (p=0.30)		Lower (p=0.0004)
**Chinese or British Chinese**	Increasing (0.13)	0.015 to 0.25	Yes (p=0.031)	0.39	Increasing (0.10)	0.074 to 0.13	Yes (p<0.0001)	0.87	Yes (p=0.58)		Higher (p<0.0001)
**Mixed**	Increasing (0.26)	0.064 to 0.46	Yes (p=0.015)	0.47	Increasing (0.17)	0.14 to 0.20	Yes (p<0.0001)	0.94	Yes (p=0.31)		Higher (p<0.0001)
**White**	Decreasing (-0.70)	-1.18 to -0.23	Yes (p=0.0081)	0.52	Decreasing (-0.23)	-0.62 to 0.16	No (p=0.22)	0.15	Yes (p=0.10)		Higher (p<0.0001)
**Other**	Increasing (0.70)	-0.11 to 0.24	No (p=0.39)	0.074	Increasing (0.18)	0.14 to 0.22	Yes (p<0.0001)	0.89	Yes (p=0.19)		No difference (p=0.22)

### Gender

**Figure 1:**
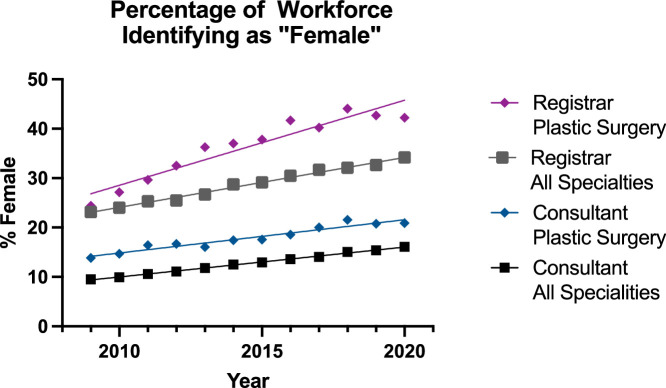
Regression for Percentage Workforce Identifying as Female in All Subgroups.

Both Plastic Surgery groups had a higher percentage of females than their all surgical specialty counterparts, with the highest percentage in the plastic surgery registrar group. The increase from 2009 to 2020 for specialty trainees was 23% to 42% (plastics) and 23% to 34% (others) and for the consultants it was 14% to 21% (plastics) and 10% to 16% (others). The percentage of female consultants and specialty trainees increased in a significant (p<0.0001) linear fashion for all groups (Figure 1 and Table 1), with a very good fit on a linear model (0.91-0.99).

The plastic surgery consultants group had a significantly higher percentage of females than the all surgical specialities consultant group (p<0.0001). The rate of increase (slope) of female clinicians was the same in the consultant groups (p=0.27). The plastic surgery registrar group had a higher percentage of females than the all surgical specialties group, and because the rate of increase (slope) was significantly higher (p=0.007) in the plastic surgery group, the significance test could not be performed.

### Less-Than-Full-Time

**Figure 2:**
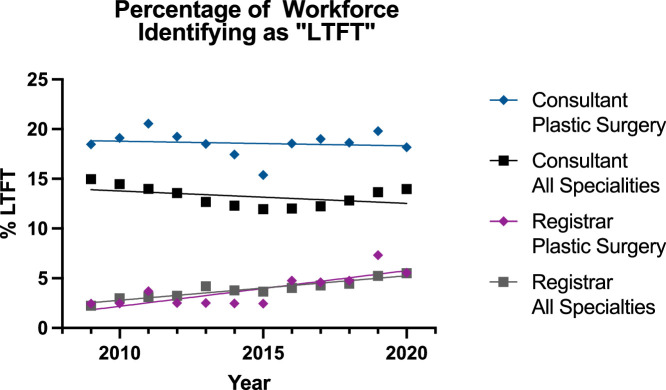
Regression for Percentage Workforce Identifying as “Less-Than-Full-Time” in All Subgroups.

The percentage of LTFT clinicians was higher in the consultant groups than that in the registrar groups (Figure 2). The trend for the period 2009-2020 was decreasing for both consultant groups (Table 1), but the linear regression model was unsuitable and a poor fit. The plastic surgery consultant group had a significantly higher percentage than the all specialties group (p<0.0001). For the registrar groups, the trend was increasing with the linear regression model being suitable and a good fit for both (p=0.0017, p<0.0001). The Registrar groups increased simultaneously (p=0.22) and there was no difference in the percentages (p=0.74). Moreover, LTFT has a positive impact on workforce well-being, patient safety and attrition, with support from the General Medical Council (GMC); however, barriers still exist and this is evident from the data.

**Figure 3:**
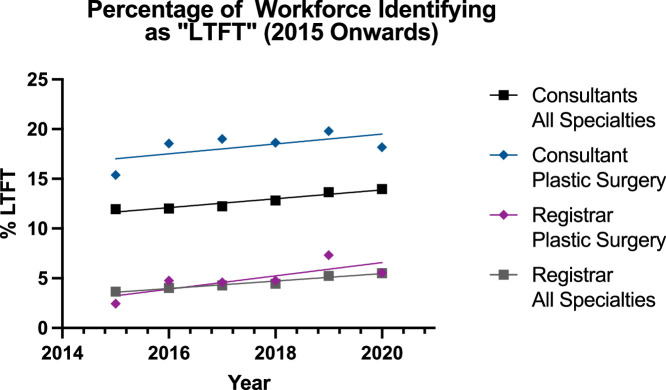
Regression for Percentage Workforce Identifying as “Less-Than-Full-Time” in All Subgroups 2015 onwards.

On visual review of the graph (Figure 2), a potential upward trend was observed from 2015 onwards. Accordingly, the data were analysed again from 2015 onwards (Figure 3). This change did not affect the trends and relations for registrar groups, resulting in the linear model not being a significantly suitable model for plastic surgery registrars (p=0.06). For the consultants, the trend for both groups was increasing, but the plastic surgery group could not be modelled significantly with a linear regression (p=0.19) unlike the all surgical specialities group (p=0.0019). Despite this, the rate of increase was not different between the two groups (p=0.88) and the plastic surgery consultants group had a significantly higher percentage of LTFT surgeons (p<0.0001).

### Ethnicity

The ethnicity data were divided by ethnicity categories and compared between specialities by grade (Table 1).

**Figure 4:**
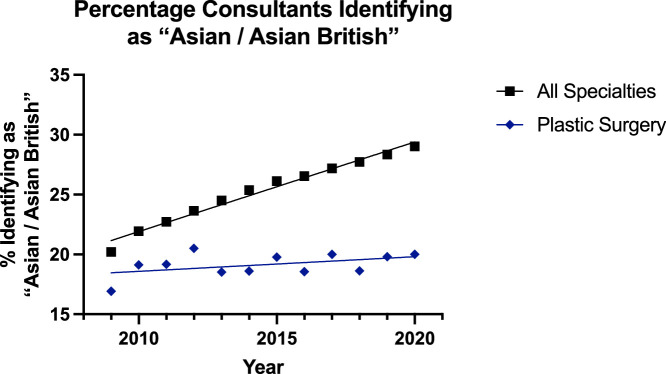
Data and Linear Regression for the Percentage of Consultants Identifying as “Asian / Asian British”.

**Figure 5:**
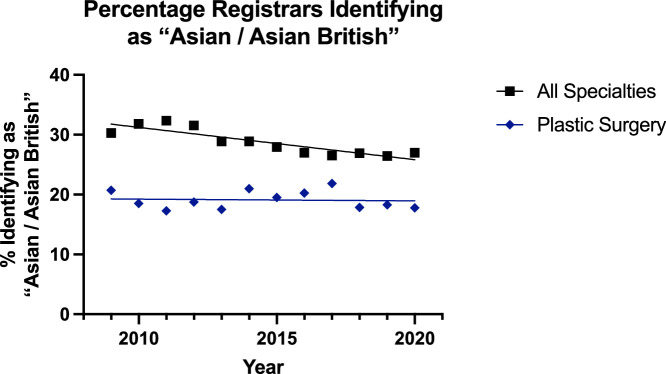
Plot of Data and Linear Regression for the Percentage of Registrars Identifying as “Asian / Asian British”.

For surgeons identifying as ‘Asian or Asian British’, an increasing trend was observed for consultants and declining for registrars in both groups (Figures 4, 5). However, the plastic surgery groups could not be significantly modelled using linear regressions, whereas the all surgical specialties groups could be. The consultant all surgical specialties group had higher elevation and rate of increase than its plastic surgery counterpart (p<0.0001). For registrars, the all surgical specialty group was decreasing at a significantly higher rate (p=0.0046) but had a higher percentage of ‘Asian/Asian British’ trainees.

**Figure 6:**
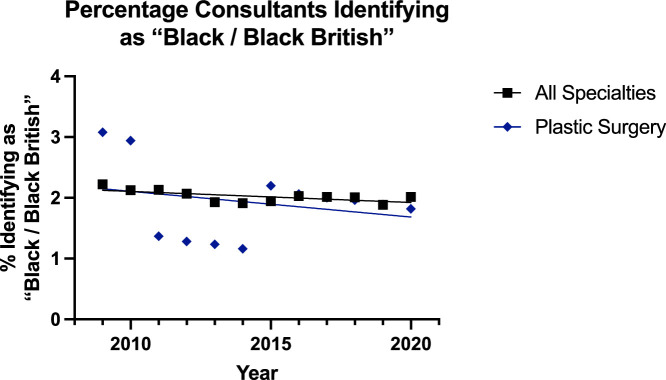
Plot of Data and Linear Regression for the Percentage of Consultants Identifying as “Black / Black British”.

**Figure 7:**
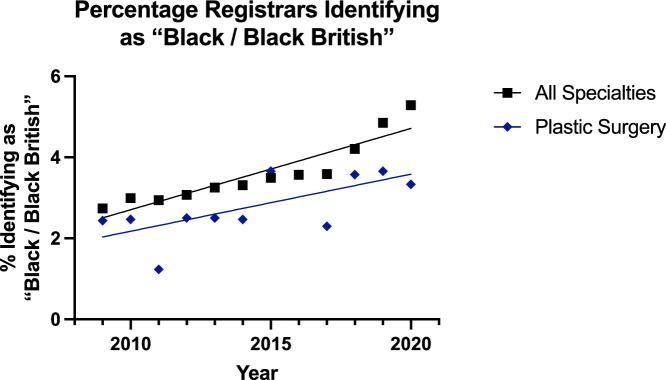
Plot of Data and Linear Regression for the Percentage of Registrars Identifying as “Black / Black British”.

With ‘Black or Black British’ surgeons, the trend appeared to be slightly decreasing at the consultant level and increasing at registrar level (Figures 6, 7). For the consultants, the linear regression model was suitable for all surgical specialties (p=0.021) but not suitable for plastic surgery (p=0.64). However, the rate was the same and there was no difference in elevation. At the registrar level, the linear regression model fit both plastic surgery (p=0.0154) and all surgical specialties (p<0.0001). The rate of increase in both groups was the same (p=0.30), but the plastic surgery group had a significantly lower proportion (p=0.0004).

**Figure 8:**
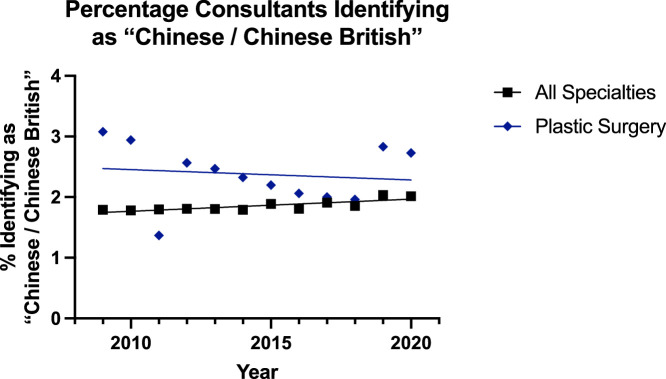
Plot of Data and Linear Regression for the Percentage of Consultants Identifying as “Chinese / Chinese British”.

**Figure 9:**
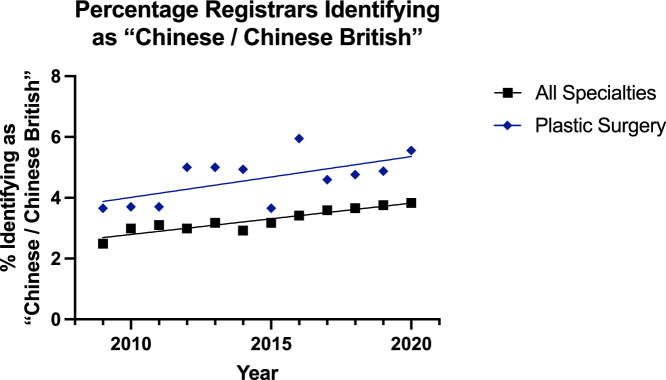
Plot of Data and Linear Regression for the Percentage of Registrars Identifying as “Chinese / Chinese British”.

The trend was increasing for all groups identifying as ‘Chinese / British Chinese’ except the plastic surgery consultant group that had a small decline (Figures 8, 9). This pattern was also noted for modelling the regression with it being suitable for all surgical specialties consultants (p=0.001), all surgical specialties registrars (p<0.001) and plastic surgery registrars (P=0.0031), but not for plastic surgery consultants (p=0.70). In the consultant groups, there was no significant difference in the trend over the time period; however, the plastic surgery group showed a significantly higher (p=0.002) difference. For registrars, this same was true of the rate (p=0.58) and plastic surgery being higher (p<0.0001).

**Figure 10:**
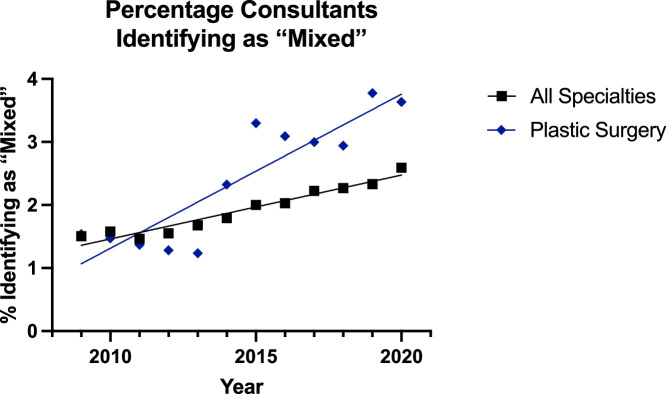
Plot of Data and Linear Regression for the Percentage of Consultants Identifying as “Mixed”.

**Figure 11:**
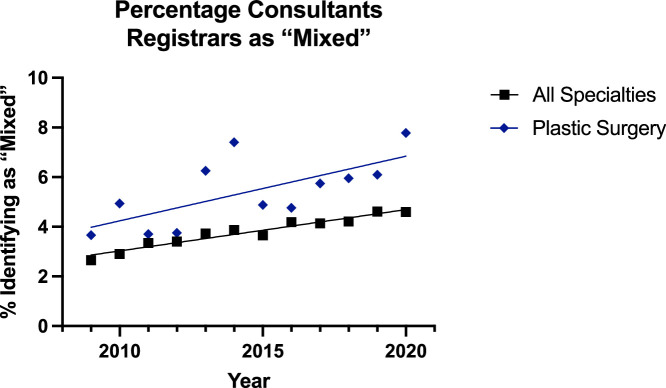
Plot of Data and Linear Regression for the Percentage of Registrars Identifying as “Mixed”.

For surgeons identifying as ‘mixed’, the trend was increasing in all groups (Figures 10, 11). Additionally, linear regression model was significantly suitable for all groups. The plastic surgery consultant group was increasing at a greater rate than the all surgical specialties group (p=0.0015) and was higher—but elevation could not be assessed as difference in slope was too high. In the registrar group, there was no significant difference between the rate of elevation, but in the plastic surgery group it was higher (p<0.0001).

**Figure 12:**
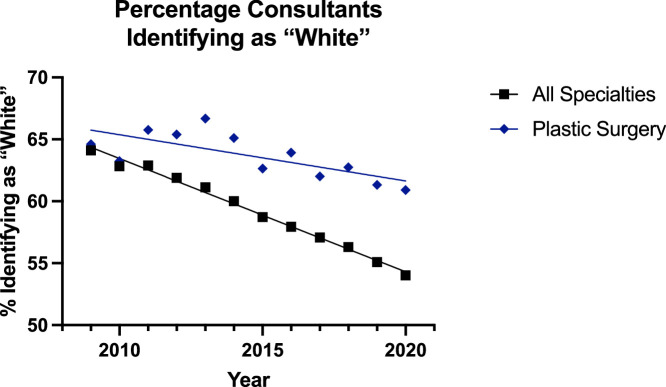
Plot of Data and Linear Regression for the Percentage of Consultants Identifying as “White”.

**Figure 13:**
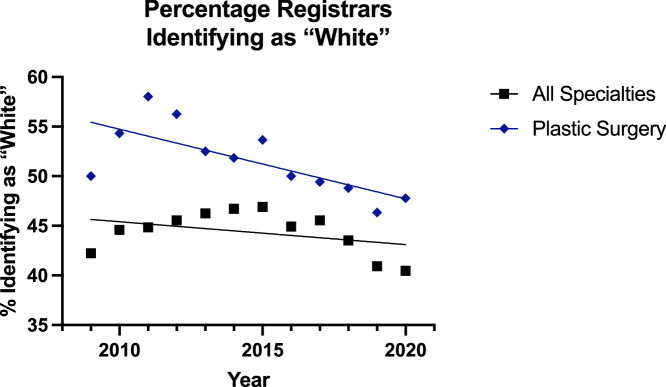
Plot of Data and Linear Regression for the Percentage of Registrars Identifying as “White”.

In instances where surgeons identified as ‘White’, the trend was decreasing across all groups (Figures 12, 13). The linear regression model was significantly suitable for all groups except for the all surgical specialty registrar group (p=0.22). The plastic surgery consultant group was decreasing at a slower rate than its counterpart (p=0.0001), and it had a greater percentage of surgeons identifying as ‘White’—but the elevation could not be assessed for significance as the difference in slope was too great. Among the registrars, there was no significant difference in the slope between the two lines, but there were significantly more trainees identifying as ‘White’ in the plastic surgery group (p<0.0001).

**Figure 14:**
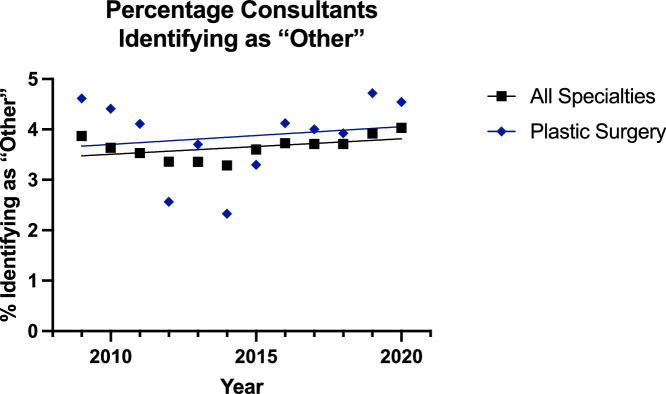
Plot of Data and Linear Regression for the Percentage of Consultants Identifying as “Other”.

**Figure 15:**
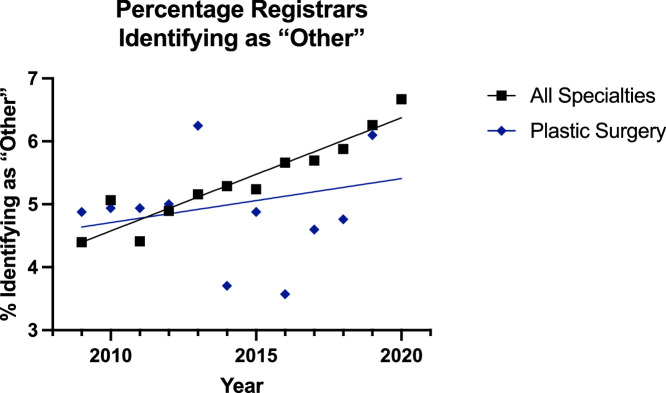
Plot of Data and Linear Regression for the Percentage of Registrars Identifying as “Other”.

Finally, for the surgeons identifying as ‘Other’, the trend was increasing in all groups (Figures 14, 15), but the linear regression model was only suitable for the all surgical specialty registrar group (p<0.0001). Between the consultants, there was no difference in the rate or elevation of the slopes; this was also true for the registrars.

## Discussion

This study provides the first in-depth analysis of the changing reconstructive plastic surgery (herein denoted as ‘plastics’) workforce in the UK within the NHS; notably, this excludes surgeons in the private, aesthetic or cosmetic sector. This data, derived from national sources, offered a comprehensive and reliable picture from 2009 to 2020 and the opportunity to assess how the sub-specialty compares to an aggregate of other surgical specialties. The overall results from this study are encouraging and show an increasingly diverse workforce, but it also identifies some points that merit further investigation, discussion or change.

Mirroring general trends in medicine for an increasing proportion of females in the workforce,^7^ the percentage of surgeons identifying as female increased in all the groups. This culminated in an increase until 2020 for specialty registrars from 23% to 42% (plastics, M:F 1.5:1) and 23% to 34% (others, 2:1), and for the consultants was 14% to 21% (plastics, 4:1) and 10% to 16% (others, 5.3:1). The reconstructive plastic surgery subgroups were persistently and significantly higher than the other surgical specialties over the period assessed (Figure 1 and Table 1). Furthermore, although the rate of increase was the same in the consultant groups, the rate of increase in the reconstructive plastic surgery registrar group was significantly higher. These increases mirrored similar increases in the proportion of female medical students and doctors in the same period—achieving gender parity in the wider profession.^8^ The percentages for specialty registrars may be reassuring when considering that in 2018, 41% of core surgical trainees were female.^9^ Assuming that the rise in this proportion continues, extrapolation of graphs suggests that reconstructive plastic surgery can possibly achieve gender parity for registrars by 2028 (assuming an identical rise in general surgery—which has similar female: male ratios).^10^ However, significant work remains to be carried out to increase the proportion of female consultants—this is being actively targeted by the Royal College of Surgeons.^11^ This initiative forms part of a wider international drive to improve gender parity in senior positions in surgery as American research shows that the proportion is markedly lower for programme directors, published research, conference presentations and academic positions as well.^12^ Although the attrition of female surgical registrars has been noted in meta-analyses elsewhere,^13^ it is historically attributed to ‘lifestyle choices’^13^ which modern surgical training can help overcome with more flexible and inclusive working practices. Thus, the proportion of female consultants can be expected to significantly increase over the next five years, if our analysis also showed improvements in flexible working—which it does with regard to LTFT.

The results for LTFT were more complex within the time period (Figure 2-3 and Table 1). Despite there being more LTFT consultants than registrars, the proportion appeared to be decreasing for both groups (plastics 18.5% to 18.2%; others 15.0% to 14.0%) from 2009-2020, but the linear regression model was unsuitable and a poor fit. When the analysis was conducted again for 2015-2020, the trends in both the consultant groups were now increasing (plastics 15.4% to 18.2%, others 11.9% to 14.0%) but the linear regression model remained unsuitable for the plastic surgery consultant group while becoming suitable for the all surgical specialties cohort. However, there were consistently more reconstructive plastic surgery consultants who were LTFT and the rate of change between the two groups in either time periods was similar. The rates in 2020 for reconstructive plastic surgery consultants was similar to that for consultants overall in the NHS—with 20% being LTFT in 2020.^14^ This may surprise some readers who recognise the potential for private practice in reconstructive plastic surgery in the UK. However, it appears the converse may be true, as this trend of increase in LTFT is matched by a downward trend in percentage of consultants who partake in private plastic surgery practice.^15^

For specialty registrars, over the full period (2009-2020), there was a significant increase that was of equal rate and amplitude in both groups (plastics 2.4% to 5.6% and others 2.2% to 5.5%). Although one can speculate that there is typically greater flexibility in the consultant workplan than that of the specialty registrar, more work is needed to understand these differences. Notably, some studies showed that up to three-quarters of medical graduates are considering LTFT work.^16^ Although half of the reconstructive plastic surgery registrars are considering LTFT,^17^ only 7% of the trainees surveyed in 2020 were LTFT—matching the 7.3% figure obtained in 2019 for reconstructive plastic surgery registrars in our analysis. Other authors have found that the three main factors prohibiting LTFT are reductions in pay, extension of training time and concerns about senior negative perceptions.^17^ The same study also found that 90% of LTFT trainees in this time period were female, and suggested that a potential reason is gender bias against male trainees who may wish to go LTFT.^17^ This forms of a wider issue in surgery internationally, with gender bias in attitudes towards aspects such as parental leave, where less paternal leave are taken and females are told their career is incompatible with parenthood.^18^

Despite this, overall in surgery, there has been a push towards achieving greater diversity, inclusion, and equity as championed by the Royal College of Surgeons of England.^19^ The results of this study showed a trend towards greater diversification of the reconstructive plastic surgery workforce, but with some areas potentially requiring review. The proportion of consultants identifying as ‘Asian or Asian British’ in reconstructive plastic surgery was both significantly lower and increasing at a slower rate that of the other group (Figure 4)—17% to 20% for plastics and 20% to 29% for others. For specialty registrars, there was a gradual decline in the proportion of this subgroup for all surgical specialities (30% to 27%) and minimal change for plastic surgery (21% to 18%)—which was significantly lower (Figure 5). Similar trends were noted for consultants identifying as ‘Black or Black British’ with the trend slightly declining for both plastic surgery (3.1% to 1.8%) and all surgical specialty groups (2.2% to 2.0%) [Figure 6]. For registrars, however, there was a significantly consistent and similar increase in the proportion of ‘Black or Black British’ trainees, but the proportion was lower in the plastic surgery group (2.4% to 3.3%) compared to the others (2.7% to 5.3%) [Figure 7].

Notably, there was a separate category of ‘Asian or Asian British’ for surgeons identifying as ‘Chinese or Chinese British’. Here, the trend was increasing for all surgeons (1.8% to 2.0%) except reconstructive plastic surgery consultants for whom there was a small decline (3.1% to 2.7%) [Figure 8]—although the linear regression model was not suitable for this group. For the registrars, the trend was significantly increasing for all specialties (2.5% to 3.8%) and plastic surgery (3.7% to 5.6%) but the trend for plastics was significantly higher (p<0.0001). These significant increases were also noted in the ‘Mixed’ category, where all subgroups saw a significant increase and the plastic surgery consultant (1.5% to 3.6%) and registrar (3.7% to 7.7%) groups were significantly higher than their other consultant (1.5% to 2.6%) and registrar (2.7% to 4.6%) counterparts.

These increases were matched with a decrease in the proportion of surgeons identifying as ‘White’ in all groups (Figures 12,13). The rate of this decline was slower in the plastic surgery consultant group (65% to 61%) than the all surgical specialty equivalent (64% to 54%) [p=0.0001], and the proportion of ‘White’ surgeons was also significantly higher in the plastic surgery registrar group (50% to 48%) compared to the other specialities (42% to 40%). Meanwhile, in the ‘Other’ group the trend appeared to be increasing in all groups (Figures 14, 15) but the linear regression model was only suitable for all surgical specialty registrars.

In light of these findings, it is apparent that the composition of the surgical workforce, particularly within reconstructive plastic surgery, is diversifying at a notable pace. The shifts observed in various ethnic categories are testament to this transition. However, a closer look at these trends reveals some disparities, as the proportional representation of certain ethnic groups such as ‘Asian or Asian British’ and ‘Black or Black British’ in reconstructive plastic surgery remains lower than that in all surgical specialties. These discrepancies underscore areas of potential concern that require further exploration and targeted efforts to address this issue. In 2020, a Royal College of Surgeons of England article called the lack of ethnic diversity in senior and leadership positions in surgery an ‘uncomfortable truth’.^20^ It cited that although racism in medicine is a ‘pervasive challenge’, the first step is to identify the trends—as we have done in this study—to challenge any biases that we identify in our workplaces.^20^

Conversely, the uptick in the representation of the ‘Chinese or Chinese British’ and ‘Mixed’ groups, coupled with a decline in the proportion of surgeons identifying as ‘White,’ shows the changing demographic fabric of the surgical profession. Despite these encouraging signs of progress, further work is necessary to ensure equity and inclusivity across all ethnic groups in all surgical specialties. This includes the need to investigate and address the underlying factors that may contribute to these trends and the disparities they highlight. Notably, the figures and graphs denoting this results appear similar and more diverse than the figures for ethnic groupings of students who booked the University Clinical Aptitude Test (entry exam for medical students) in the same period: White (69%), Indian (10.2%), Pakistani (4.8%), mixed (4.2%), other Asian background (3.9%), Black (2.9%), other ethnic background (2.2%), Chinese (1.6%) and Bangladeshi (1.1%).^21^

Accordingly, the challenge is to understand where the increased diversity among registrars and consultants originates from, and whether the diversity arises from local or international recruitment. It is likely that this diversity came from UK medical graduates rather than from international graduates as work conducted by the General Medical Council suggests that most international medical graduates (IMGs) enter the workforce in junior, non-training posts^22^—although the proportion of IMGs in training programmes has subsequently increased. ^23^ Notably, data on the proportion of IMGs in formal plastic surgery training programmes and as consultants—except from studies from the US showing their limited participation and representation— are limited.^24^^,^^25^ Therefore, it becomes probable but uncertain that the increase in ethnicity and diversity at a consultant level occurs owing to increases in diversity at the specialty registrar level. The same is also true of the specialty at the registrar level, where this increase follows the increased diversity in UK medical schools. However, more research is required as there is inadequate literature on IMGs in UK reconstructive plastic surgery, and it is possible that IMGs could be entering the training programmes following time in non-training roles such as junior clinical fellowships.

### Limitations

Although this study provides a thorough overview of workforce trends within the UK's surgical specialties, it is not without its limitations and it is specific only to the UK.

Firstly, the data used in this study were based on NHS Digital's workforce data, which, although reliable, is subject to the accuracy of the data inputted at source. Next, the study uses broad categories for gender and ethnicities, which may not accurately capture the full spectrum of identities or ethnicities within the surgical workforce—so to oversimplify findings. Further yet, only data prior to 2020 was analysed and so recent changes or trends are not reflected in the study. Additionally, the study used linear regression models, which may have not been suitable for more complex underlying trends—e.g. changes secondary to legislation or event. Finally, although this study presents a detailed overview of the workforce trends in surgical specialties, it does not explore the underlying reasons. Therefore, it is necessary to interpret the results within the larger context of societal, institutional and individual factors that influence career choices and progression within the surgical profession. Future research should explore these factors in more depth to better understand and address the observed trends.

## Conclusion

This investigation delivers an analysis of the demographic evolution within reconstructive plastic surgery in the UK. The results of our research underscore the growth in gender and ethnic diversification, alongside varying trajectories in the adoption of LTFT work patterns within this sphere. These data suggest an on-going transformation towards a more diverse surgical workforce, although the pace of this transformation varies among surgical specialties and grade. For all trends, we need to identify at what point the change occurs, entry to medical school, foundation year, core training (or equivalent) or specialty training and what can be done.

Nonetheless, despite these encouraging transitions suggestive of a more inclusive discipline, observable disparities remain among different groups, particularly concerning ethnicity and LTFT work tendencies. These disparities may warrant further exploration to maximise access and inclusivity within the profession.

## Declaration of competing interest

None.
